# Paramyxovirus Glycoprotein Incorporation, Assembly and Budding: A Three Way Dance for Infectious Particle Production

**DOI:** 10.3390/v6083019

**Published:** 2014-08-07

**Authors:** Farah El Najjar, Anthony P. Schmitt, Rebecca Ellis Dutch

**Affiliations:** 1 Department of Molecular and Cellular Biochemistry, University of Kentucky College of Medicine, Lexington, KY 40536, USA; E-Mail: farah.najjar@uky.edu; 2 Department of Veterinary and Biomedical Sciences, The Pennsylvania State University, University Park, PA 16802, USA; E-Mail: aps13@psu.edu

**Keywords:** paramyxovirus, matrix protein, glycoproteins, virus assembly, viral trafficking, membrane rafts, virus budding

## Abstract

Paramyxoviruses are a family of negative sense RNA viruses whose members cause serious diseases in humans, such as measles virus, mumps virus and respiratory syncytial virus; and in animals, such as Newcastle disease virus and rinderpest virus. Paramyxovirus particles form by assembly of the viral matrix protein, the ribonucleoprotein complex and the surface glycoproteins at the plasma membrane of infected cells and subsequent viral budding. Two major glycoproteins expressed on the viral envelope, the attachment protein and the fusion protein, promote attachment of the virus to host cells and subsequent virus-cell membrane fusion. Incorporation of the surface glycoproteins into infectious progeny particles requires coordinated interplay between the three viral structural components, driven primarily by the matrix protein. In this review, we discuss recent progress in understanding the contributions of the matrix protein and glycoproteins in driving paramyxovirus assembly and budding while focusing on the viral protein interactions underlying this process and the intracellular trafficking pathways for targeting viral components to assembly sites. Differences in the mechanisms of particle production among the different family members will be highlighted throughout.

## 1. Introduction

**Figure 1:**
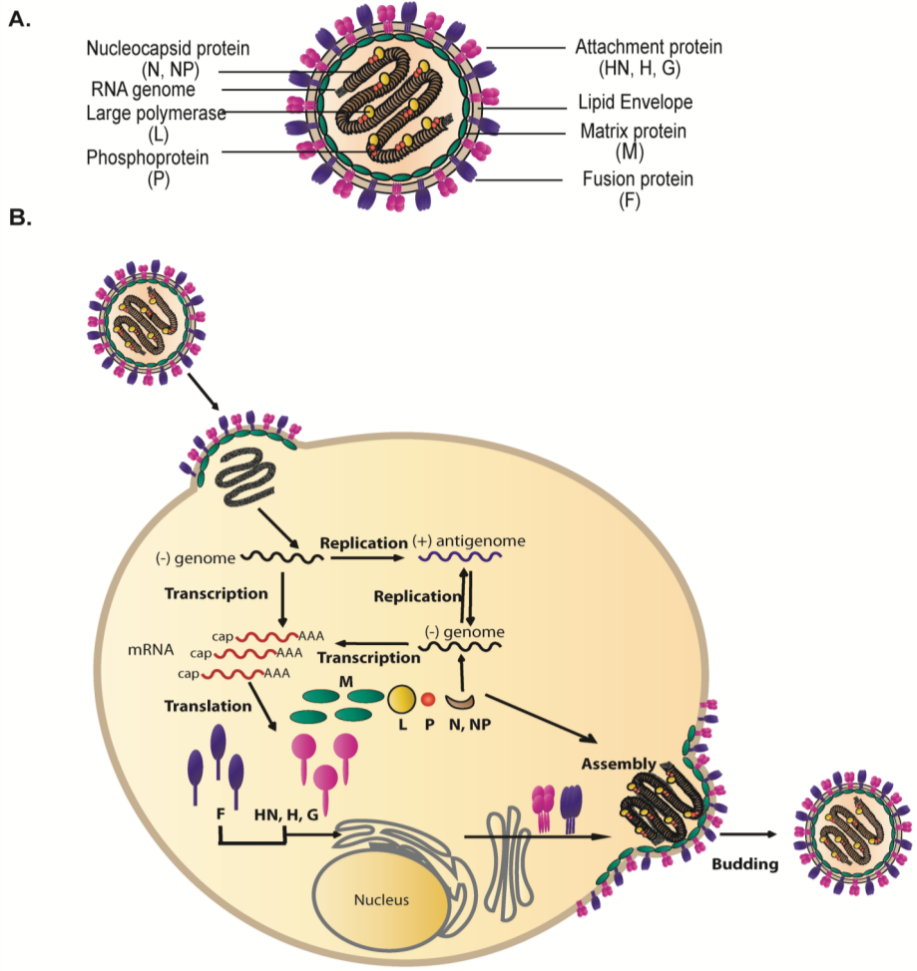
(**A**) Schematic of a paramyxovirus particle. The viral envelope, containing two main surface glycoproteins: fusion protein (purple) and attachment protein (magenta), surrounds the single stranded RNA genome (gray) which is encapsidated by the nucleocapsid protein (brown) and bound by phosphoprotein (orange) and the large polymerase protein (yellow). Underlying the membrane is a layer of matrix proteins (green). (**B**) Schematic illustration of the life cycle of paramyxoviruses. Transcription and replication of the viral genome occurs in the cytoplasm by the action of the viral RNA-dependent RNA polymerase. The newly synthesized viral components translocate to discrete sites at the infected cell plasma membrane where assembly and budding of infectious virus particles occur. For details, refer to text.

The *Paramyxoviridae*, a family of enveloped viruses with negative strand, non-segmented RNA genomes, cause significant disease in humans and animals. Important human pathogens within this family include measles virus (MeV), mumps virus (MuV) and human respiratory syncytial virus (HRSV), which is the single largest cause of respiratory tract infections in the pediatric population [1]. In addition, several paramyxoviruses have recently been identified, including the respiratory pathogen human metapneumovirus (HMPV) and the deadly zoonotic Hendra (HeV) and Nipah (NiV) viruses [2,3,4]. Paramyxoviruses also lead to high burdens on agriculture and the global economy by infecting avian species (Newcastle disease virus (NDV) and avian metapneumovirus (AMPV) [5,6], cattle (rinderpest virus), as well as pigs (NiV) [3] and horses (HeV) [4]. Paramyxovirus particles are pleomorphic in shape. For many family members, particles are primarily spherical, and range in size from 150 nm to 300 nm in diameter; however, a filamentous form predominates for some viruses such as HRSV and the parainfluenza viruses, and these particles can reach up to 10 μm in length [7,8,9,10,11,12]. The process by which paramyxovirus particles are formed and released at the cell membrane involves a series of highly coordinated and organized events that eventually result in the production of fully infectious virus particles with the basic structure depicted in Figure 1A. The viral membrane of paramyxoviruses contains two major glycoproteins required for virus entry into target cells: the attachment protein (termed HN for hemagglutinin-neuraminidase, H for hemagglutinin, or G for glycoprotein, depending on the virus) and the fusion (F) protein. These glycoproteins are densely packed on the viral envelope and form spike layers as seen under cryo-electron microscopy [13,14]. A subset of paramyxoviruses have an additional surface glycoprotein, the small hydrophobic (SH) protein whose function in the viral life cycle is less clear since it is dispensable for virus replication *in vitro* [15,16,17,18,19]. Inside the viral envelope, the RNA genome is encapsidated by the nucleocapsid proteins (N or NP), forming the flexible, loosely coiled nucleocapsid structure, termed ribonucleoprotein complex (RNP), to which the viral RNA-dependent RNA polymerase complexes, made of large polymerase (L) protein and phosphoprotein (P), are bound. The RNA genomes of paramyxoviruses are 15–19 kb in length and contain six to ten genes. As is the case for most negative-strand RNA viruses, association of the paramyxovirus RNP with the viral membrane is mediated by the matrix (M) protein. Matrix proteins are considered the key organizers of virus particle assembly since they act as bridges between the envelope glycoproteins and the ribonucleoprotein complexes, can self-assemble into higher order structures, and bind cellular membranes as well as several cellular factors [20,21,22].

Figure 1B depicts the general life cycle of paramyxoviruses, which culminates in newly synthesized virus particles being assembled and released into the extracellular matrix. Infection is initiated upon binding of the attachment protein to a cell surface receptor, followed by fusion of the viral membrane to a host cell membrane, a step promoted by the F protein. The viral genome is then released into the cytoplasm where all the steps of the replication cycle occur. Primary transcription of the negative sense RNA genome by the viral RNA-dependent RNA polymerase follows the “stop-start” model resulting in a gradient of mRNA abundance such that genes at the 3'end are transcribed in higher amounts than genes at the 5'end [1]. Replication of the full-length genome occurs efficiently only after accumulation of viral proteins and involves production of positive sense anti-genomes which act as templates for the synthesis of new negative-sense genomic RNA. Progeny genomes can then be used for further replication, for secondary transcription, or for incorporation into virus particles. The newly synthesized RNPs are then transported to selected sites at the plasma membrane where interaction with the viral integral membrane glycoproteins occurs, followed by membrane scission and release of virus particles. Incorporation of RNPs and envelope glycoproteins into infectious virus particles is a highly complex and coordinated process that requires cooperation among the three main structural components of the virus: the surface glycoproteins, the RNPs and the matrix proteins. While the majority of paramyxoviruses fit with this overall model, studies on the molecular mechanisms involved in the assembly and budding of paramyxovirus particles revealed significant differences between members of this family. This review will focus on novel findings in the understanding of the interplay between surface glycoproteins, matrix proteins and RNPs during virus particle assembly while highlighting the main differences that exist among the members of this family.

## 2. Interactions among the Viral Proteins are Critical for Glycoprotein Incorporation and Paramyxovirus Particle Assembly

The three key components in production of infectious paramyxovirus particles, the surface glycoproteins, the matrix proteins and the RNPs, must coalesce at the plasma membrane to initiate budding. Interactions among these three components are critical for glycoprotein incorporation and particle assembly. The matrix protein is generally considered the main driver of paramyxovirus assembly and can interact with both the glycoproteins and the core RNPs in an orderly manner. However, paramyxovirus surface glycoproteins are not simply bit players in this process, but instead can play important roles in directing the process of particle formation.

### 2.1. Paramyxovirus Surface Glycoproteins

Entry of paramyxoviruses into target cells requires the concerted effort of two glycoproteins on the viral membrane: the attachment protein and the F protein. The attachment protein is generally responsible for primary adsorption of the virus to the cell surface by binding proteinaceous or sialic acid receptors [23,24,25]. Fusion of the viral envelope to a target cell membrane then occurs, a process that is driven by very large conformational changes in the F protein [26,27,28]. Paramyxovirus attachment proteins are all homotetrameric type II integral membrane proteins [29], but their nomenclature differs depending on two characteristics: the ability to agglutinate erythrocytes (hemagglutination), and the presence or absence of neuraminidase activity (cleavage of sialic acid). For the genera Rubulavirus, Respirovirus and Avulavirus, the attachment protein is termed HN for its ability to cause hemagglutination (H) and remove sialic acid from carbohydrates (N). The attachment proteins of Morbilliviruses can agglutinate red blood cells but lack a neuraminidase activity and are thus denoted H. G is used to refer to the attachment proteins that lack both the ability to bind and to cleave sialic acids, which is the case for the attachment proteins of Henipaviruses and members of the *pneumovirinae* subfamily. Interestingly, the attachment proteins of pneumoviruses differ significantly from attachment proteins within the *paramyxovirinae* subfamily, as they are much smaller in size and are not strictly required for membrane fusion and entry [16,19,30,31,32,33]. Recent data strongly suggest that the F protein plays a role in attachment for members of the *pneumovirinae* subfamily, and cellular factors which interact with both RSV F and HMPV F have been identified [34,35,36,37].

**Figure 2:**
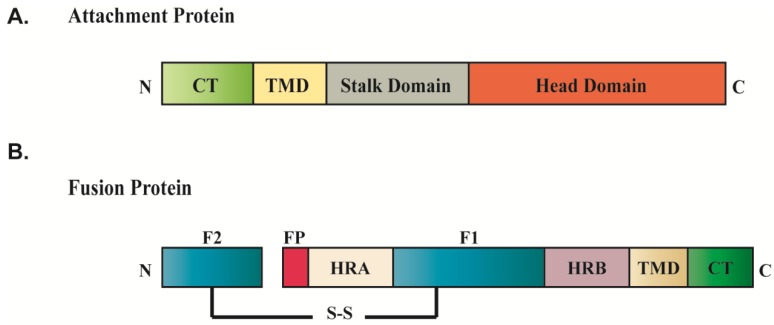
Conserved domain structures of paramyxovirus fusion protein (**A**) and attachment protein (**B**). Abbreviation: fusion peptide (FP); heptad repeat region (HRA, HRB); transmembrane domain (TMD); cytoplasmic tail (CT); disulfide bond (S-S).

Evidence from X-ray crystallography on a number of paramyxovirus attachment proteins, including the HN proteins of NDV, PIV5, and PIV3; the H protein of measles virus and canine distemper virus (CDV); and the G protein of HeV and NiV, suggests that the attachment proteins exist as a dimer-of-dimers, with each monomer comprising a short cytoplasmic tail, a single membrane-spanning domain and a large ectodomain made up of a membrane-proximal stalk region and a *C*-terminal globular head domain (Figure 2A) [38,39,40,41,42,43,44,45,46,47,48]. The crystal structures for both the stalk and the head domains have been obtained, revealing important information about the mechanistic role of the attachment protein in linking the binding of cell surface receptors to the triggering of F protein-promoted membrane fusion. The globular head, composed of four six-bladed β-propeller fold monomers, harbors the sites for receptor binding and enzymatic activity, though the exact location differs according to the virus. The atomic structures of the stalk domains of PIV5 and NDV showed that this domain forms a four helix bundle, and substantial evidence suggests that F interacts with the attachment protein through this stalk domain [42,49,50,51,52,53,54,55,56,57]. Interaction of the attachment protein with F is generally required for triggering the fusion-associated conformational changes needed for membrane fusion.

Paramyxovirus F proteins contain a fusion peptide, two heptad repeat regions, HRA and HRB, a *C*-terminal cytoplasmic tail, and a single-pass transmembrane domain which anchors the protein to plasma membrane (Figure 2B). They are homotrimeric type I proteins that are synthesized in a fusogenically inactive precursor form, termed F_0_, and require proteolytic processing into the fusogenically active, disulfide-linked F_1_+F_2_ metastable pre-fusion form [58]. Proteolytic processing of all F proteins is necessary to expose the hydrophobic fusion peptide needed for membrane insertion, but the protease responsible for the cleavage differs depending on the virus. Some paramyxovirus F proteins, such as those of HMPV and Sendai virus, are cleaved by tissue-specific extracellular proteases, so that virus assembly involves the uncleaved precursor form [2,32,59]. For most paramyxoviruses, the cleaved metastable pre-fusion F_1_+F_2_ heterodimer is the predominant form incorporated into virus particles, so cleavage must occur prior to virus assembly. For the majority of paramyxoviruses, including mumps, PIV5, and NDV, processing of F is mediated by the ubiquitous subtilisin-like serine protease furin during F protein transport through the *trans* Golgi network, prior to arrival at the plasma membrane [60,61,62]. Henipavirus fusion proteins require activation by the endosomal/lysosomal protease cathepsin L, an event that is accomplished after endocytosis of F_0_ from the cell surface and trafficking to an early endosomal compartment, followed by re-trafficking to the plasma membrane [63,64,65,66]. The HRSV F protein is unique among paramyxoviruses since it requires two cleavage events for its activation, one mediated by furin in the TGN prior to assembly, and a second cleavage which is thought to depend on cathepsin L in an endocytic compartment after endocytosis of the viral particle [67]. The F protein interacts with its homotypic attachment protein in a temporally and spatially controlled manner, though the timing of this interaction may vary among different paramyxoviruses. There is evidence that in some cases, a preassembled F/attachment protein complex is formed prior to packaging into budding virions, while in other cases, the F and attachment proteins incorporate separately into particles and only interact within the viral envelope upon subsequent receptor binding. Specific interaction between the fusion and attachment proteins during viral particle assembly will be discussed in more detail in Section 3.1. Upon receptor binding, the attachment protein transmits a signal to F which triggers a series of irreversible conformational changes in F leading to the formation of a stable six-helix bundle formed by the heptad repeat regions and resulting in fusion of the viral envelope with the host cell membrane [25,68,69,70].

Some paramyxoviruses including members of the *pneumovirinae* subfamily, rubulaviruses, and the unclassified J virus have an additional glycoprotein on the viral membrane, termed SH for small hydrophobic protein. SH proteins are all type II integral transmembrane proteins, but the size and proposed function of these proteins differs between viruses. The SH proteins of RSV and HMPV have been proposed to function as viroporins, or viral protein channels [71,72,73,74,75,76,77]. In addition, HMPV SH can modulate the host immune response and contribute to viral pathogenicity by inhibiting NF-κB [78]. For other paramyxoviruses, SH can inhibit apoptosis by interfering with TNF-α signaling [15,79]. Recent evidence indicates that HMPV SH can also decrease HMPV F-mediated membrane fusion and inhibit virus uptake in dendritic cells [77,80]. Although studies have shown that SH is dispensable for virus replication *in vitro* [17,18,19,81], deletion *in vivo* can attenuate viral replication and pathogenicity [16,30,82,83,84].

### 2.2. Matrix Proteins as Coordinators of Paramyxovirus Assembly and Budding

The M protein, the most abundant protein in the virion, plays a fundamental role in paramyxovirus assembly through its ability to interact with multiple partners. M proteins can self-assemble, bind directly to cellular membranes, and interact with the RNP complex and the cytoplasmic tails of glycoproteins, thus allowing the RNP core to associate with a region at the plasma membrane where the surface glycoproteins are concentrated, which will become the budding site. The importance of M proteins for paramyxovirus particle production was originally shown in Sendai virus and measles virus, where mutations in the M gene encoding an unstable M protein was correlated with severe defects in infectious particle production [85,86,87,88]. Our understanding of the role of M protein in the process of paramyxovirus assembly was enhanced by studies involving virus-like particle formation (VLPs) and reverse genetics, as recombinant viruses with mutations or deletions in the M gene revealed the significance of the matrix protein in incorporation of other viral components and in viral budding. For example, deletion of the measles virus M protein led to an increase in cell-associated virus and the loss of co-localization of the surface glycoproteins with the RNPs [89]. A recent study by Mitra *et al.* showed that infection with an M-null HRSV resulted in impairment of infectious particle release and alterations in the intracellular localization of the RNP complex as well as in the distribution of glycoproteins on the plasma membrane, further demonstrating the essential role of M in the assembly and budding of virus particles [90]. For many paramyxoviruses, including Sendai virus (SeV) [91,92], MeV [93,94], NiV [95,96], hPIV1 [97], and NDV [98], transient expression of M protein by itself is sufficient to promote budding of VLPs, indicating that the M protein of these viruses has the ability to efficiently associate with membranes, induce membrane curvature and promote scission. Although matrix proteins of different paramyxoviruses display similar functions, they vary greatly in length and amino acid sequences.

Recent structural studies on paramyxoviruses involving crystallography and cryo-tomography revealed important information on how the structure of M and how its organization within virions allows M to function in assembling viral components and inducing membrane deformation. The atomic structures of M proteins of three paramyxoviruses, NDV [99], HRSV [100] and HMPV [101] were recently solved. These M proteins share similar overall structures, including the presence of positive charges on the surface of the molecule which are observed in other mononegavirales matrix proteins including Ebola virus VP40 [102] and Borna disease virus (BDV) M protein [103]. Although the NDV and HMPV M proteins were crystallized as dimers while the HRSV M protein was found in a monomeric form, structural alignment revealed similarities in the N-terminal domain (NTD) and C-terminal domain (CTD) of these proteins [99,100,101]. The monomeric subunits of NDV, RSV and HMPV M proteins are composed of two beta-sheets containing folded domains that are connected by an unstructured, flexible linker region. The linker region is thought to play a dynamic role in promoting structural plasticity of M which is essential for the ability of the protein to interact with multiple binding partners and with itself. Matrix-like proteins are known for their ability to form higher order assemblies involving NTD/NTD and CTD/CTD interfaces. For SeV, it has been shown that M can self-assemble *in vitro* into helices and sheets [104,105]. Binding of HRSV and HMPV M proteins to lipids promotes self-assembly and polymerization of M subunits into long flexible helical filaments with different curvatures [101,106]. It is thought that the dimer subunits of M can associate through different side-by-side interactions which influence that curvature of the matrix arrays and thus virus morphogenesis. Paramyxovirus M proteins, similar to matrix proteins of other negative-strand RNA viruses, also have an intrinsic ability to bind membranes [107,108,109,110,111], and the matrix protein of NDV was even shown to adsorb onto phospholipid liposomes and self-assemble to induce membrane deformation [112]. However, the exact nature of the interaction of M with membrane lipids is not yet clearly understood. The atomic structures of the paramyxovirus M proteins are characterized by the presence of a positively charged area on the surface of the molecule, most likely involved in electrostatic interactions with the negatively charged surface of cell membranes. Binding of M to the lipid bilayer is suggested to be driven by basic residues in the CTD as well as by hydrophobic interactions [100]. Interestingly, comparison of amino acid sequences of pneumovirus M proteins revealed high similarity in the CTD and more disparate sequences in the NTD, suggesting further that the *C*-terminal interactions of different paramyxovirus M proteins with membranes are of a similar nature and that the NTD is involved in specific protein interactions of M with other viral and cellular factors [100]. This would thus explain the ability of various paramyxovirus M proteins to coordinate different protein-lipid and protein-protein interactions. The matrix protein of HMPV has an additional unique feature in its NTD not seen in other mononegaviral matrix proteins to date which is the presence of a Ca^2+^ binding site [101]. This suggests that binding of Ca^2+^ to HMPV M may represent a new mechanism by which M proteins of paramyxoviruses are regulated to organize particle assembly.

Despite the essential role of the M protein in paramyxovirus particle production, the mechanisms by which M regulates the assembly and budding processes vary among different members of the family. Unlike SeV, MeV, NDV, NiV and hPIV3, where the M protein is sufficient for VLP formation, other paramyxoviruses require interactions of M with the surface glycoproteins or with the RNP for particle formation, indicating that there are significant variations in the function of M and in the strategies that different family members employ for efficient particle production. Differences in the role of M in the assembly of paramyxoviruses were also demonstrated by electron cryo-tomography showing the 3D structures of virus particles. While the general ultrastructural model of paramyxoviruses depicts M protein as lining the inner leaflet of the viral envelope, recent cryo-tomography data show that this structure does not apply to all paramyxoviruses. For RSV, NDV and SeV, M forms a layer under the viral membrane only in a small percentage of virus particles. In the majority of particles, M was observed to be dissociated from the membrane and disassembled, potentially to allow the conformational changes of the F protein from the pre-fusion to the post-fusion form by releasing interactions with the F cytoplasmic tail [99,113,114]. A recent study revealed that for RSV, the surface area of the virion membrane which is covered by M varies significantly depending on the morphology of the virus particle, with the highest coverage (86%) detected in filamentous particles and the lowest (24%) in spherical viruses [115]. The arrangement of the surface proteins and the matrix proteins in the 3D structures of NDV and RSV suggest an interaction between these two viral components. In MeV, on the other hand, M protein was not located under the viral membrane but was found to assemble on the RNP forming a bundled two-layer helical structure inside the virion [116]. These findings suggest significant mechanistic differences in the way M interacts with the RNPs and envelope proteins to assemble virus particles.

### 2.3. Interaction of Ribonucleoprotein Complex with Glycoproteins and M

During replication, the newly synthesized genomic RNA is tightly wrapped with the nucleoprotein for protection from degradation, forming a helical RNP complex [117]. Encapsidation of RNA by N does not depend on specific nucleotide sequences, as expression of N in the absence of infection can result in the formation of nucleocapsid-like structures resulting from N non-specifically binding host-cell RNAs [92,97,118,119,120]. Prior to virus budding, newly synthesized RNPs must assemble with the surface glycoproteins and the M protein at the plasma membrane. While multiple copies of the RNA genome can be packaged within a single particle [114,115,121,122], incorporation of RNPs into virions is selective and likely depends on species homology between M and the nucleocapsid protein, genome length, and to a lesser extent on the polarity of the genome [123,124,125]. Targeting of RNPs to the plasma membrane assembly sites is primarily mediated by the M protein. M proteins of several paramyxoviruses, including SeV, MeV and PIV5, are known to interact with the nucleocapsid protein to mediate incorporation of the RNPs into virions [123,126,127]. Studies using recombinant viruses also demonstrated that deletions or mutations of the M gene can block RNP complex transport to the plasma membrane during infection, further supporting the important role of M protein in RNP inclusion into virus particles [90,94]. Within the pneumovirinae subfamily, association of M with the RNP can occur through interaction of M with the transcription elongation factor, M2-1 protein, which is also considered a component of the RNP complex [113,115,128]. M can also bind RNA directly or can bind to the large polymerase L protein [129,130]. In addition to interacting with M to facilitate their incorporation into virions, in some cases the paramyxovirus nucleocapsid proteins play a role in increasing efficiency of VLP budding [14,119]. Co-imunoprecipitation experiments showed that the fusion protein of NDV interacts with the NP protein in purified VLPs and not with the M protein, suggesting that interaction of F with NP may be involved in localization of NP at plasma membrane assembly sites [98]. In other cases, such as SeV, interaction of the M protein with a viral glycoprotein is required for concentration of the RNPs at the plasma membrane and their subsequent assembly into particles [131].

### 2.4. Active Role of Glycoproteins in Paramyxovirus Particle Formation

While the role of M proteins as organizers of paramyxovirus assembly has been well established, the important function of membrane proteins in the late phases of paramyxovirus infection has gradually become clearer. Surface glycoproteins of paramyxoviruses are well characterized for their significance in membrane fusion and viral entry; however, substantial evidence implicates an active role of these membrane glycoproteins in the end stages of the virus replication cycle (reviewed in [20,21]). Paramyxovirus glycoproteins can specify the location for viral budding through interactions with lipids, associate with the M protein to aid in assembly, and in some cases, interact with RNPs as part of virus assembly. For assembly of infectious particles, M must target the cytoplasmic RNPs to the budding site at the plasma membrane where the viral integral membrane glycoproteins are concentrated, thus paramyxovirus M proteins are suggested to bind membranes at areas enriched with the envelope proteins. Consistent with this view, the ultrastructure of NDV revealed that the M protein forms a grid-like array where the glycoproteins were densely packed [99]. In addition, an inner layer of membrane-bound M was associated with regular spacing of the pre-fusion F protein in RSV virions, further supporting an interaction between M and F [113]. Studies have demonstrated that membrane proteins interact with the matrix protein for a number of paramyxoviruses, and this interaction is needed to organize assembly and for the incorporation of glycoproteins into budding virus particles, but many differences exist between various members with respect to the contribution of this interaction to particle formation and the individual roles of the attachment and fusion proteins. For SeV, M can interact with both F and HN [132,133,134] but only the fusion protein is important for virus production and its function is as critical as that of M since alterations in F can attenuate virus production up to 70% [134,135,136]. Expression of glycoproteins was also shown to be important for budding of VLPs. For PIV5, F and HN have redundant functions during VLP formation, while mumps F protein, but not HN, enhance particle release [14,119,137,138]. Loo *et al*. have recently shown that while HMPV M interacts with both F and G proteins, expression of HMPV G facilitates formation of VLPs [10]. Specific interactions between M and HN of NDV have also been reported; however, this interaction does not have an effect on efficiency of VLP release [98]. Similar results were seen for MeV, NiV and RSV, indicating that glycoproteins can enhance budding efficiency for only a subset of paramyxoviruses [93,95,96,139].

In addition to their contribution to the budding process, paramyxovirus glycoproteins are also implicated in assembly of other viral components. The fusion protein of RSV was shown to be responsible for incorporation of G and SH proteins into budded virions and for their co-localization with N at plasma membrane assembly sites; however, F deletion had no effect on M assembly into virions [139]. A key function for the fusion protein in SeV assembly was shown, as mutations in F altered cellular localization of both HN and M, although interaction of F with M was not affected [131]. These findings indicate that the paramyxovirus glycoproteins can play significant roles in the assembly and budding processes, but different paramyxoviruses utilize their glycoproteins differently.

### 2.5. Role of the Cytoplasmic Tails of Glycoproteins in Particle Production

**Figure 3:**
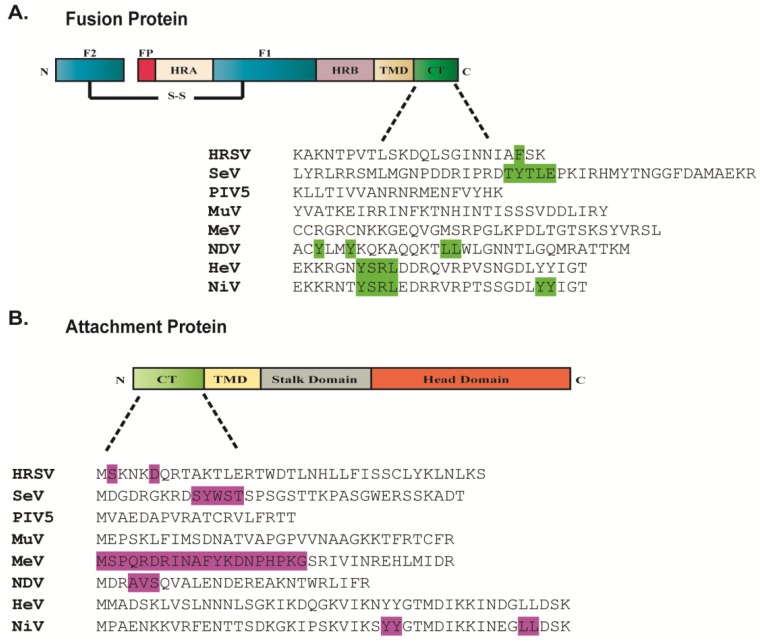
Amino acid sequences of the cytoplasmic tails of the fusion protein (**A**) and attachment protein (**B**) of different paramyxoviruses. Amino acid residues highlighted contribute to paramyxovirus particle production. Sequences of cytoplasmic tails were obtained from UniProt.

Paramyxovirus surface glycoproteins contain short cytoplasmic tails which extend on the inner side of the plasma membrane. The length and amino acid sequences of these domains in the fusion and attachment proteins vary significantly among different paramyxovirus members (Figure 3). Several studies have demonstrated that the role of paramyxovirus glycoproteins in particle formation depends on their cytoplasmic tails, as these regions are required for glycoprotein incorporation into packaged particles and for glycoprotein interactions with M. Biochemical and co-localization studies revealed that M can interact with the cytoplasmic tail of the homotypic attachment proteins for RSV, HMPV, NDV and measles [10,98,140,141]. These finding are surprising for the pneumoviruses RSV and HMPV since G is dispensable for viral replication *in vitro* [30,31]. This suggests that while interaction of M with G is dispensable for virus production in these cases, the presence of G may contribute to optimal virus production manifested by an increase in HMPV VLP formation [10] and a role for G in SH incorporation into RSV particles [139]. The importance of the glycoprotein cytoplasmic tails for paramyxovirus particle production was also demonstrated for PIV5, as deletion of these domains in either F or HN prevented assembly of M and the glycoproteins at the cell surface, and removal of the cytoplasmic tail of the HN protein significantly decreased virion egress [119,138]. For other paramyxoviruses, the cytoplasmic tail of F protein plays an important role in late stages of viral infection. The cytoplasmic tails of MuV and hPIV1 F proteins are involved in particle assembly [14,142]. For RSV, formation of viral filaments depends on the cytoplasmic tail of the fusion protein [139,143]. In addition, alterations in the cytoplasmic tail of SeV F protein significantly affected virus replication and the clustering of HN, M and NP at assembly sites, indicating a role for the cytoplasmic tail of the fusion protein in coordinating the assembly of the different viral components [131,142]. For measles virus, truncations in the cytoplasmic tails of F and H do not alter assembly of viral components at the cell membrane but do affect the incorporation of F, M and H into released particles [144]. Deletion of the cytoplasmic tail of measles virus F protein is associated with increased cell-cell fusion, similar to what is seen for the MeV strain obtained from subacute sclerosing panencephalitis (SSPE) patients. It has been suggested that interaction of M with the cytoplasmic tails of F locks F in the pre-fusion conformation during the process of assembly; thus removal of the cytoplasmic tail, and loss of the M interaction domain, facilitates fusion [89,113,145]. The importance of the glycoprotein cytoplasmic tails for particle assembly appears to be a common feature of many RNA viruses, as truncations in the cytoplasmic tails of the glycoproteins hemagglutinin [146] (HA) and neuraminidase (NA) of influenza A virus or of the rhabdovirus G result in severe defects in particle formation [147,148]. Figure 3 shows the differences in the length and amino acid sequences of the cytoplasmic tails of different paramyxovirus glycoproteins. It is believed that the function of the cytoplasmic tails of glycoproteins in paramyxovirus particle formation depends on specific amino acid sequences or signals rather than simply a defined length. The sequences that have been shown to affect the role of the fusion and attachment proteins in particle production are highlighted [91,131,140,143,144,149,150,151,152,153,154]. For SeV, it was shown that while random truncations and mutations in the cytoplasmic tail of F affected virus production to varying extents, the strongest effect on accumulation of virus components at the cell surface and virus egress was detected in particles carrying F proteins with mutations in a TYTLE motif [91,131]. A four amino acid sequence with a critical phenylalanine residue in the cytoplasmic tail of HRSV F protein was shown to coordinate virus filament assembly and budding [143,146]. Specific sequences crucial for the insertion of attachment proteins into virions have also been identified for NDV and SeV HN proteins (Figure 3B) [149,153]. These findings suggest that even though the cytoplasmic tails of paramyxovirus glycoproteins are needed for their inclusion into infectious viruses, the roles of these domains in different viruses may vary.

## 3. Intracellular Trafficking of Viral Components

During paramyxovirus replication, the glycoproteins, matrix proteins and RNPs are synthesized at distinct sites in the cytoplasm and must be transported to the plasma membrane for coordinated assembly. The different viral components reach the plasma membrane by different mechanisms and interact with each other in an orderly manner either during trafficking or at the cell surface prior to packaging into virions. Paramyxovirus proteins are carried to the cellular plasma membranes by utilizing various cellular machineries including endocytic and exocytic pathways, in addition to vesicular trafficking and the cytoskeleton.

### 3.1. Trafficking of Viral Glycoproteins

Paramyxovirus glycoproteins are synthesized in the endoplasmic reticulum (ER) and traffic through the secretory pathway, and in some cases through endocytic pathways, to the plasma membrane. Proper trafficking is needed for incorporation into budding virions or induction of cell-cell fusion for direct cell-to-cell transmission of virus particles. For some paramyxoviruses, data indicate that the fusion and attachment proteins can interact following their synthesis in the ER, and thus are transported to the cell surface as a metastable protein complex. This has been suggested to occur for NDV, MeV and human parainfluenza viruses 2 and 3 [155,156,157]. Alternatively, the F protein and the attachment protein can traffic separately and only associate after reaching the plasma membrane, which is the case for HeV and NiV [158,159]. For PIV5, F and HN also associate at the cell surface but formation of the F-HN complex requires receptor binding [68]. During their synthesis in the ER, glycoproteins must undergo proper folding and oligomerization prior to trafficking to the cell surface. Mutational analyses showed that mutations which interfere with proper folding or assembly of the final oligomeric structure of paramyxovirus glycoproteins generally result in their retention in the ER and prevent their transport through the exocytic pathway to the cell surface [160,161,162,163]. The contribution of the ectodomain in proper folding and stability of the trimeric fusion protein or the tetrameric attachment protein is well established, but substantial evidence also indicates an important role of the transmembrane domains and cytoplasmic tails in the oligomerization process and folding of the ectodomain [29,162,164,165]. Mutation of a TYTLE motif in the cytoplasmic tail of SeV F protein prevented its transport to the PM, and the protein was instead retained in the ER. This failure to traffic was hypothesized to be due to the failure of the F mutant to trimerize [131]. Similar findings were reported for the PIV5 HN protein, as deletion of the cytoplasmic tail prevented its assembly to an oligomer and transport to the cell surface [163]. In addition to their role in protein oligomerization, the cytoplasmic tails are thought to facilitate proper trafficking of glycoproteins to the cell surface by binding cellular factors that direct protein targeting to the plasma membrane and by harboring residues that facilitate interaction with negatively charged lipids at the plasma membrane. *N*-glycosylation can also be essential for the proper folding, stability, intracellular transport, and surface expression of the paramyxovirus glycoproteins. Removal of *N*-glycans from the glycoproteins of NDV, CDV, PIV5, SeV, HeV and NiV had a significant effect on their exocytic transport and surface expression [166,167,168,169,170,171,172]. However, removal of all three *N*-glycans did not affect transport of the RSV F protein to the cell surface indicating that the degree to which *N*-glycosylation influences proper folding and transport varies among paramyxovirus glycoproteins [173,174].

Trafficking of viral glycoproteins has been demonstrated to involve tyrosine-based and di-leucine motifs which are involved in protein trafficking in both secretory and endocytic pathways. Several paramyxovirus glycoproteins have endocytic signals and can undergo internalization following trafficking to the plasma membrane [65,175,176,177,178]. For instance, the cytoplasmic tails of NiV and HeV F proteins contain a tyrosine-based motif (YXXΦ), where X is any amino acid and Φ is a residue with a bulky hydrophobic side chain that is required for internalization of the protein from the cell surface [65,178]. PIV5 HN is internalized from the plasma membrane by clathrin-coated pits but its internalization depends on a single glutamic acid residue at the boundary between the transmembrane domain and the ectodomain [176,179]. Henipavirus fusion proteins are the only paramyxovirus glycoproteins that have an absolute dependence on endocytosis for proteolytic activation by cathepsin L [63,65,66,180]. With the exception of Henipaviruses, the relevance of endocytic signals in viral envelope glycoproteins is not yet well established. It has been proposed that down-regulation of attachment and fusion protein expression on the cell membrane may be a post-translational regulatory mechanism that plays an important role in viral pathogenicity through minimizing recognition of antigens on the infected cells by the immune system. Endocytic signals in viral glycoproteins can also affect efficiency of glycoprotein incorporation into virions and virus assembly. Mutation of the internalization signal in PIV5 HN has been shown to affect the incorporation of both F and HN into budded virions [181]. Interaction of the surface glycoproteins with the core matrix proteins may regulate expression of the paramyxovirus glycoproteins on the cell surface and decrease internalization of the glycoproteins, thus favoring their incorporation into assembled virus particles over endocytosis [89,145,182,183].

Paramyxoviruses can infect cells that are polarized in nature, such as neurons, epithelial cells, endothelial cells and lymphocytes. The plasma membrane in polarized cells is divided into two discrete domains, the apical domain and the basolateral domain, and this polarity is maintained by sorting of proteins and lipids in the TGN or secretory pathways and recycling endosomes [184]. Several studies conducted on paramyxoviruses in polarized systems have revealed important information about the sorting of the surface glycoproteins between apical and basolateral sides. Motifs for internalization and polarized targeting generally share common elements that can interact with specific adaptor complexes, such as the cytoplasmic adaptor proteins, and thus selectively recruit protein cargo into endosomes, lysosomes or target them to basolateral cell membrane [185]. Recognition of two separate signals for endocytosis and basolateral sorting may be regulated by interaction with other viral components. NiV glycoproteins expressed from plasmid DNA are located primarily at the basolateral surface of epithelial cells, and this localization depends on tyrosine signals (YXXΦ) in the cytoplasmic tail of Nipah F and on a dityrosine signal in the Nipah G protein [150]. The F and H proteins of measles virus also have tyrosine-dependent sorting signals in the cytoplasmic domains that mediate their targeting to the basolateral site of polarized epithelial cells and facilitate cell-cell fusion between epithelial cells. Transport of measles F and H is not interdependent but each protein traffics alone, as mutation of tyrosines in one protein does not affect localization of the other [186,187]. Additional motifs in cytoplasmic domains that direct basolateral targeting have been identified in non-viral systems, and include the tetrapeptide NPXY motif, dileucine motifs and single leucine residues [188,189]. Fitting with this, a dileucine motif in the cytoplasmic tail of the NDV F protein was shown to be important for the basolateral sorting of the protein [152]. A role for the TMD in intracellular transport of integral membrane proteins is being increasingly recognized. For example, amino acid residues S490 and Y498 in the TMD of Hendra virus F protein were found to be critical for endocytic trafficking and recycling of the protein to the cell surface [154,164,190]. The hypothesis that the localization of surface glycoproteins determined the site of virus budding was dominant for a considerable time, but substantial evidence indicates that this is not accurate in many cases. Directional budding is determined by the matrix protein for several enveloped viruses, including influenza, Marburg virus and VSV [191,192,193,194]. The paramyxovirus M protein is also considered the main determinant of virus budding sites. Crucial evidence for the central role of M in determination of budding sites was obtained from studies on MeV and NiV, where experiments showed that while the glycoproteins are intrinsically targeted to the basal side of polarized membranes, budding of virus particles occurs at the apical side where the M protein is concentrated, and expression of M during infection partially redirects the glycoproteins to apical surface [150,186,187,195,196,197]. Basolateral sorting of the fusion and attachment proteins can contribute to the pathogenesis of the virus by mediating transmission of viruses to underlying tissues by cell-cell fusion. For RSV, both the M protein and the glycoproteins are targeted to the apical side of polarized epithelial cells, but the glycoproteins are dispensable for apical sorting of M and for virus budding at the apical side, further confirming the essential role of M in directionality of budding [198]. Apical targeting of membrane proteins depends on signals either in extracellular domains or in the TMD. For example, the TMDs of influenza HA or NA are necessary for polarized sorting of the two proteins [184,199,200]. The TMD of RSV fusion protein also plays a role in the polarized sorting of the protein to the apical side [201]. Apical targeting can also be established by alternative mechanisms. Association of proteins with lipid rafts (cholesterol and sphingolipid rich domains) can facilitate transport of protein cargo to apical membranes, and raft association has been shown for several paramyxovirus glycoproteins as well as for several paramyxovirus M proteins [202]. This will be discussed in more detail in the following section. Signals for basolateral sorting are usually dominant over apical signals, so retargeting of paramyxovirus glycoproteins from the basal to the apical side during infection requires masking of basolateral targeting signals [203,204,205]. Transcytosis from the basolateral to the apical side, which requires endocytosis from the specific domain, delivery to endosomes, and trafficking to the apical surface can also occur, and involves apical recycling endosomes (ARE). Sorting of the M protein and glycoproteins of measles to different membrane domains during infection indicates that trafficking of M occurs independently of the surface proteins. This is consistent with the finding that the matrix protein of measles is not co-transported with glycoproteins [109]. However, for some paramyxoviruses such as SeV [206], M associates with the glycoproteins before reaching the plasma membrane. For RSV, M and F were found to localize in inclusion bodies (IBs) in the cytoplasm for the formation of assembly complexes prior to trafficking to the surface of nonpolarized cells. Deletion of the cytoplasmic tail of F altered the cellular localization of both proteins, with both M and F found concentrated in inclusion bodies and not in filaments on the cell surface. Although a direct interaction between RSV M and F has not been shown, F and M targeting to the plasma membrane requires the cytoplasmic domain of F [146]. Interestingly, RSV F and M were shown to traffic to the apical side independent of one another suggesting that polarized sorting of viral proteins can vary significantly from non-polarized trafficking.

### 3.2. Intracellular Transport of Matrix Proteins and Ribonucleoproteins

Though originally synthesized in the cytoplasm, the matrix protein and the RNPs must subsequently translocate to viral budding sites at the plasma membrane; however, very little is currently known on the mechanisms underlying transport of these critical viral structural components. In the classical model for paramyxovirus assembly, the matrix protein is thought to interact with the RNP at the cell membrane to mediate its insertion into budding sites for the production of infectious virus particles. Substantial data, however, support an alternative model in which the matrix protein associates with the RNP complex in the cytoplasm prior to translocation to the plasma membrane. Data on both MeV and SeV suggest that the M protein binds to the RNP in the cytoplasm, and the two components are then co-transported to the plasma membrane [94,108]. Further support for an interaction of M with the RNP in the absence of membrane interactions was provided by the 3D structure of MeV particles, which showed that measles M protein did not form a layer underneath the viral envelope, but instead associated with the RNPs to form a helical matrix-covered nucleocapsid structure inside the virion [116].

Data from live cell imaging revealed an important role of the host cytoskeleton in the trafficking of paramyxovirus RNP complexes. Filamentous RNPs of RSV show myosin-motor driven directional movement on the actin cytoskeleton [207]. In the case of SeV and MeV, RNPs are transported along microtubules using Rab11A containing vesicles, key regulators of trafficking within the recycling endosomal pathways and Golgi to the plasma membrane [208,209]. Rab11 endosomes are also part of the apical recycling endosome (ARE) pathway which controls apical transport of proteins in polarized cells, suggesting that this pathway may be particularly important in polarized cells. However, a requirement for Rab11A in assembly of SeV is observed in both polarized and non-polarized cells. In contrast, Rab11A dependent transport of measles RNPs is only critical for virus production in polarized epithelial cells and is not a general requirement for measles RNP trafficking. The Rab11-mediated recycling pathway is also important for budding of RSV particles from the apical surface [210]. In the course of RSV infection, the matrix protein localizes in cytoplasmic bodies containing the RNP complex proteins N, P, L, and M2-1, which are thought to be assembly bodies. Deletion of the matrix gene prevents the translocation of the viral RNP from the cytoplasmic inclusions to the cell surface suggesting that for RSV, trafficking of the RNPs depends on trafficking of M [90,211]. These findings indicate that large differences exist in the trafficking mechanisms of paramyxovirus RNPs. It is yet to be determined whether the Rab11 mediated pathway is utilized by other paramyxoviruses to transport the RNPs prior to assembly and if M is associated with the viral RNPs in the Rab11 containing endosomes to facilitate its trafficking. A recent study demonstrated that the incorporation of HIV1-Env protein into budding particles is dependent on the interaction of the Rab11-interacting proteins FIP1C/RCP and Rab14 with the cytoplasmic tail of the protein [212]. This raises the question of whether sorting of paramyxovirus glycoproteins to the plasma membrane can be mediated by components of the Rab11 pathway and this requires further investigation.

Although the entire replication cycle of paramyxoviruses occurs in the cytoplasm, the matrix proteins of HRSV, SeV, NDV and NiV have been shown to traffic through the nucleus early during virus infection. In the case of RSV, localization of M in the nucleus occurs through interaction of a nuclear localization signal (NLS) with the nuclear import receptor, importin β1, and its exit to the cytoplasm at later stages of infection is mediated by a nuclear export signal (NES) that directs Crm-1dependent nuclear export [213]. Nuclear-cytoplasmic trafficking of NiV M was also dependent on a NLS and a leucine-rich NES [214]. In contrast to both RSV and NiV, the matrix protein of NDV is present in the nucleus throughout infection, and recent studies indicate that NDV M localizes in the nucleolus primarily due to interaction with the nucleolar phosphoprotein B23 [215,216,217,218,219,220]. In all cases, trafficking of M to the nucleus and its localization there was necessary for later virus budding and efficient virus production. Although more studies are needed to clarify the biological function of M protein nuclear localization, it is proposed that M transits to the nucleus at early stages of infection to allow optimal transcription and translation of viral components, since the M protein of several paramyxoviruses has been shown to bind RNA directly and inhibit viral transcription [126,130]. Transition of M through the nucleus may also affect host transcription to enhance virus replication (similar to the matrix protein of vesicular stomatitis virus) [221,222]. The M protein of RSV has been shown to induce cell cycle arrest in lung epithelial cells by regulating cellular p53 expression levels, thus enhancing virus replication [223].

## 4. Localization of Glycoproteins at Membrane Assembly Sites: A Role of Raft Microdomains

Paramyxoviruses have a host-derived, lipid bilayer envelope containing the membrane spanning envelope glycoproteins. The assembly of viral membranes is a sophisticated and specific process that involves coalescence of viral components at discrete sites on cellular membranes and exclusion of the majority of host cell membrane proteins. Recent data support the idea that viral glycoproteins of enveloped RNA viruses are not randomly distributed on the cell surface but instead are clustered within lipid raft membrane microdomains to form the nucleation points for budding [224]. Lipid rafts, which are rich in cholesterol and sphingolipids, are characterized by a rigid, ordered structure with limited flexibility and can thus act as platforms for virus assembly (reviewed in [21,225,226,227,228]). Lipid rafts can also be referred to as detergent resistant membranes (DRM) since they are resistant to solubilization by cold non-ionic detergents such as Triton-X100. The important role of lipid rafts in assembly and budding of enveloped RNA viruses have been described for HIV-1, influenza virus, Ebola virus and others [228,229,230,231]. Several studies have reported that different paramyxovirus glycoproteins are selectively targeted to raft microdomains in cellular membranes. For MeV, only F, but not H, has the intrinsic ability to be incorporated into membrane rafts, and it is thought that two palmitoylated cysteines in the TMD of F facilitate its interaction with lipid rafts; however, during infection, H is pulled into raft domains upon association of the H-F complex with membrane rafts [232,233,234]. On the other hand, both SeV glycoproteins, F and HN, can associate with rafts when expressed individually or in combination [235]. During NDV infection, F and HN interact with rafts to facilitate the incorporation of F-HN complexes into virions [236]. The fusion protein of RSV can also be sorted to lipid rafts in a process directed by its cytoplasmic tail [237]. In addition to the glycoproteins, other viral components can be associated with raft domains in membranes. For example, the nucleoproteins of SeV, MeV and NDV have been found to associate with DRMs [233,236,238]. Matrix proteins can also bind raft membranes, in some cases regardless of the presence of other viral proteins, as was seen with the M protein of MeV [93] , and in other instances, like SeV M for example [132], depending on interaction of M with surface glycoproteins. It has been recently proposed that accumulation of viral components at cell membranes leads to coalescence of multiple membrane microdomains where viruses create their own assembly platforms [224,239]. In the case of influenza virus, these clusters of membrane microdomains are termed viral “budozones”. They are larger in size than regular cellular raft membranes and their formation is dependent on the M1 matrix protein [225,229,240,241]. It would be of interest to explore whether such domains can be formed during paramyxovirus assembly and whether their formation depends on clustering of envelope glycoproteins or on oligomerization of the matrix proteins underlying the plasma membrane and driving the multimerization of the membrane glycoproteins. In addition to acting as sites of assembly, raft domains also contribute to the infectivity of the released viral particles. Disruption of raft microdomains by altering cholesterol levels leads to a decrease in the formation of infectious particles of NDV and RSV [236,242]. For MeV and SeV, rafts are needed as platforms for assembly but do not constitute precursors for budding [232,243]. Thus, although targeting of viral components to raft membranes seems common among paramyxoviruses, the functional significance of these microdomains in the viral life cycle varies among the family members.

## 5. Role of Envelope Glycoproteins in Paramyxovirus Budding

Budding of enveloped viruses is a complex process that requires induction of membrane curvature followed by membrane scission and release of virus particles. Induction of membrane curvature and the final membrane fission event requires manipulation of the lipid-lipid interactions within cellular membranes, and is driven by interactions of viral proteins with membrane lipids in addition to viral-viral and viral-host protein interactions [244]. The mechanisms underlying budding of paramyxoviruses are still largely unknown, but it is evident that various paramyxoviruses exit infected cells using different mechanisms (reviewed in [20,21]). Budding of paramyxovirus particles is driven primarily by the matrix protein. The M protein binds membranes and homo-oligomerizes underneath the plasma membrane to drive membrane deformation and promote the needed curvature. As previously mentioned, the M protein of a number of paramyxoviruses can induce formation of VLPs when expressed by itself [91]. In this case, self-association of M under the membrane may be sufficient to drive membrane deformation and outward budding of VLPs. It is equally possible that host proteins are recruited by M to the plasma membrane and thus the host machinery drives the membrane deformation and outward budding. One of the primary mechanisms involved in the release of nascent virus particles of many enveloped viruses, such as HIV-1, Ebola virus, and VSV requires a short stretch of amino acids in the matrix protein with a late budding function known as the “L” domain. These L domains, which vary among different viruses (P[T/S]AP, PPxY, YxxL), function by recruiting and interacting with cellular proteins of the endosomal sorting complex required for transport (ESCRT), which are part of the vacuolar protein sorting (VPS) pathway and are involved in promoting membrane fission steps that lead to the release of virus particles [245]. The paramyxoviruses PIV5 [119], NDV [220] and mumps virus [14] rely on the host ESCRT machinery during virus exit, as release of particles was inhibited by expression of a dominant negative VPS4A. Budding of PIV5, NDV and mumps virus is dependent on a FPIV-like motif in the M protein [14,220,246], which does not resemble canonical L-domain sequences, suggesting that these paramyxoviruses may utilize different components of the host ESCRT that can recognize and bind to a different amino acid sequence. There is increasing evidence that a growing number of viruses, including influenza virus and VSV, can bud from host cells independent of ESCRT machinery (reviewed in [244,247]). Budding of RSV [210], NiV [248], MeV [249], AMPV [250] and HMPV [251] has also been demonstrated to occur in an ESCRT-independent manner. The mechanisms used by ESCRT-independent viruses to bud from infected cells are still unknown for many of these viruses. Interestingly, budding and virus release of HRSV is dependent on Rab11-FIP2, and the Rab11 pathway was also shown to play a role in influenza virus production, suggesting that viruses may utilize the Rab11 endosomal pathway in a previously uncharacterized manner to achieve their exit from host cells [210,252].

Although M proteins are considered the driving force for budding of paramyxoviruses, increasing evidence demonstrates that the glycoproteins can also play a role. Several paramyxovirus glycoproteins can induce VLP formation by themselves or must be present with the M protein for efficient VLP formation, indicating that surface glycoproteins in these cases are needed either to recruit M to assembly sites or to initiate budding. A major role of glycoproteins in paramyxovirus budding is well characterized for the SeV fusion protein. Sendai F induces VLP release when expressed alone in cells, and silencing of the F gene reduces virus production by 70% [134]. The ability of F to bud from the plasma membrane depends on a TYTLE motif in the cytoplasmic tail of the protein [131,253]. This suggests that the TYTLE motif may be needed to bind a cellular factor that facilitates budding. Sendai F also interacts with M in the ER and is responsible for carrying M to the plasma membrane. Interestingly, both proteins were found to contain amino acid sequences that resemble actin binding domains [91]. The host cytoskeleton has been shown to play an important role in the life cycle of several paramyxoviruses, and it is thought that cytoskeletal components are involved in paramyxovirus budding. Large amounts of actin were found associated with SeV particles and, interestingly, mutations of the actin binding domain in F resulted in a significant reduction in SeV VLP production, indicating that binding of SeV F protein to actin is important for budding of the virus. The requirement of the cytoplasmic tail domains of glycoproteins for budding of several paramyxoviruses may indicate that these domains are involved in binding cellular factors that usually are involved in exocytic pathways. Another significant role of paramyxovirus glycoproteins in paramyxovirus particle production is manifested in RSV production. Short filament-like structures containing F and G were seen in cells infected with M-null virus suggesting that RSV glycoproteins are capable of deforming the cell membrane and initiating bud formation [90]. Clustering of glycoproteins in lipid raft microdomains may create a pulling force on the plasma membrane and thus induce an initial membrane deformation that is further elongated by oligomerization of the matrix protein [113]. These observations suggest that the glycoproteins of paramyxoviruses can actively contribute to the budding process leading to virus egress from infected cells.

## 6. Concluding Remarks

**Figure 4:**
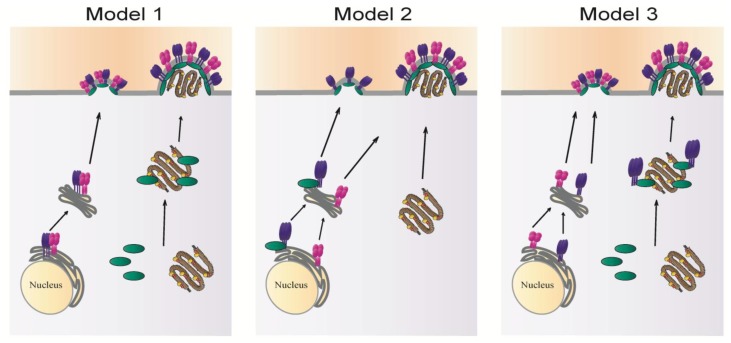
Potential models of paramyxovirus assembly: fusion protein shown in purple, attachment protein in magenta, matrix protein in green and the RNP complex in brown.

Substantial progress has been made in understanding the mechanisms of paramyxovirus particle production. Paramyxoviruses form at selected sites at the plasma membrane of host cells as a result of coordinated interactions between viral components and between viral and cellular factors. The role of the matrix protein as the principal organizer of the assembly process has been well established, and recent structural data obtained from several paramyxovirus matrix proteins revealed important information on the molecular basis of the ability of M to drive particle formation. M can associate with the plasma membrane, self-oligomerize to form a lattice, and interact with glycoproteins, the RNP complex and several host factors mainly through its *N*-terminal domain. The viral protein-protein interactions involved in particle assembly have been demonstrated for a variety of paramyxoviruses. While there are differences between members of the family in terms of specific host cell protein interactions or points of viral protein-protein interactions, the general concepts of coordinated assembly are consistent. However, the mechanisms by which different viral components reach the plasma membrane and coordinate their localization at assembly sites still require further investigation. It is evident from the studies summarized in this review that fundamental differences exist among the different family members in the mechanisms that underlie coordinated targeting to the assembly site, and three different models for paramyxovirus assembly can at present be deduced (Figure 4). What factors determine which mechanism is employed by specific paramyxoviruses for completion of their life cycle is currently unknown. It is possible, however, that some family members can employ more than one assembly mechanism either simultaneously or at different times during infection, depending on cellular factors or *in vivo* conditions.

In the first model, the fusion and attachment proteins interact following their synthesis in the ER and are co-transported to the plasma membrane as a complex. The matrix protein associates with the RNP in the cytoplasm and carries it to the plasma membrane where it assembles with surface glycoproteins. This model can be deduced mainly from studies done on MeV. Alternatively, the fusion and attachment proteins can traffic separately to the cell surface. In some cases, like SeV (model 2), the fusion protein can bind the matrix protein in the ER, and the two are transported as a complex to the plasma membrane where they create a nucleation site for assembly. Incorporation of the attachment protein likely occurs though interactions with M or with F. The RNP can traffic by itself to the assembly site and is packaged within particles upon binding to M or one of the glycoproteins. Studies of RSV suggest a third model of paramyxovirus assembly, where the formation of an assembly complex containing F, M and the RNP core occurs in inclusion bodies in the cytoplasm, with a role of the cytoplasmic tail of F in targeting M-RNP to assembly sites. While these models, and potentially others to be generated after future research, provide insight into glycoprotein incorporation and paramyxovirus assembly, several critical questions remain to be answered to obtain a complete understanding of paramyxovirus particle formation.

One of the key unanswered questions is how are assembly sites initiated? Is the clustering of surface glycoproteins in membrane raft domains sufficient to create an outward bud in the plasma membrane to which other viral components are recruited, or is the interaction of M with the cytoplasmic tail of glycoproteins and its self-oligomerization the main driver for the formation of assembly nucleation sites? The requirements for the formation of budding precursor sites may vary among different paramyxoviruses. For RSV, the fusion protein seems to be the significant contributor for the formation of short viral filaments without the need for M or the host cytoskeleton. On the other hand, the actin cytoskeleton and the interaction between M and the cytoplasmic tail of F appear to drive SeV particle formation at the plasma membrane. Another significant area of study is to determine the cellular pathways that are utilized by the matrix proteins and the RNP core that allows their delivery to assembly sites and subsequent packaging of the RNA genome into virions. Important questions also remain regarding how membrane budding and the final scission process are established for paramyxoviruses, particularly for those viruses that do not utilize the well-characterized ESCRT proteins. What are the cellular factors that play a role in budding of paramyxoviruses, and how are they recruited by viral proteins? Thus, further studies are still required to clarify multiple aspects of paramyxovirus particle production and to uncover the differences that exist in the molecular mechanisms utilized by different paramyxoviruses to form new infectious particles. Future discoveries in this field may contribute to the development of antivirals against paramyxovirus infections.
